# Supplementary material for: Modeling Aceria tosichella biotype distribution over geographic space and time
Source: PLoS One. 2020 May 29;15(5):e0233507. doi: 10.1371/journal.pone.0233507 (PMC7259573; doi:10.1371/journal.pone.0233507)
Supplement: S1 Fig — (PPTX) [file pone.0233507.s001.pptx]

## Slide 1
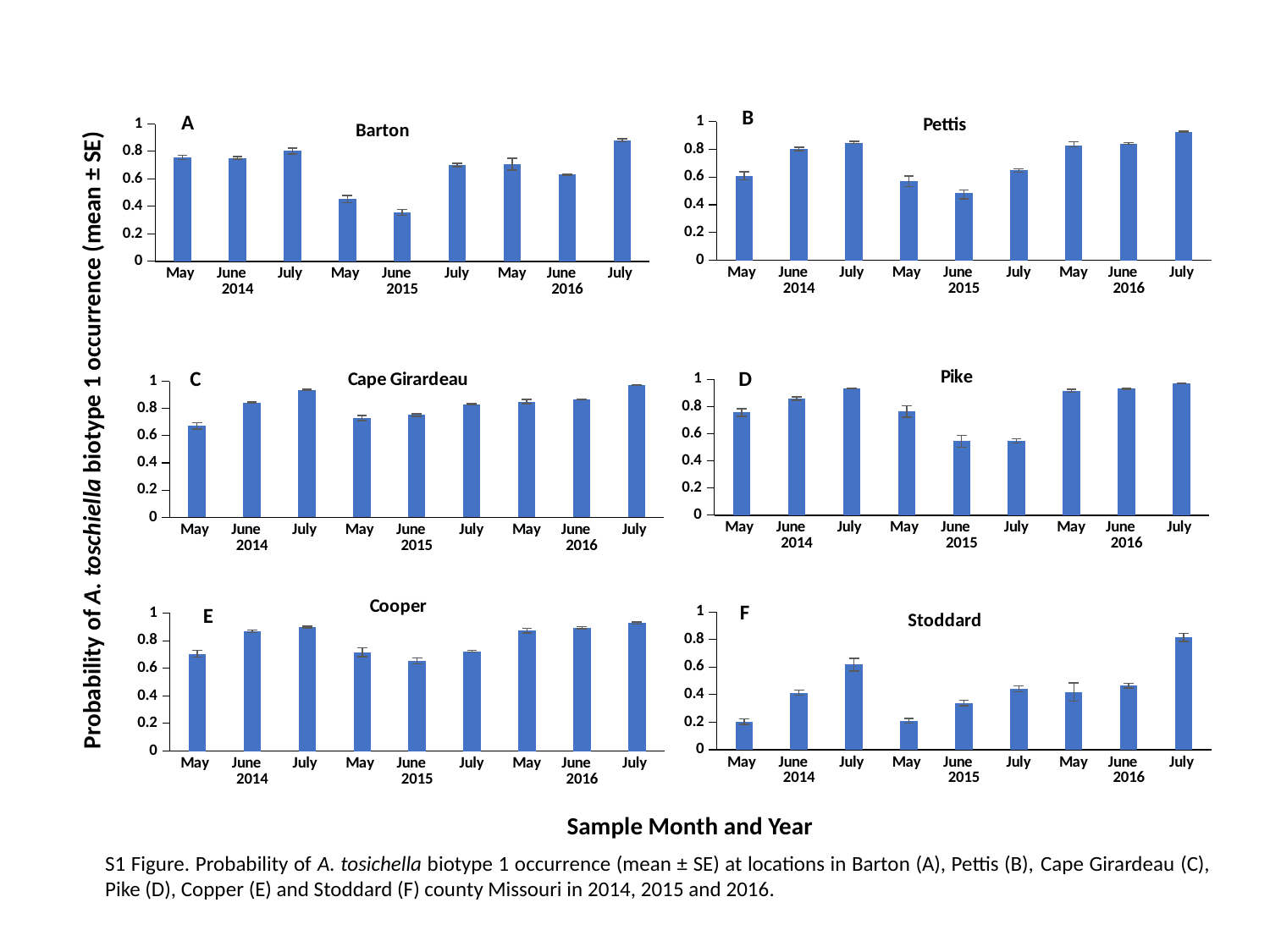

### Chart: Pettis
| Category | Pettis |
|---|---|
| May | 0.60844179359017 |
| June 2014 | 0.802063745986896 |
| July | 0.845350325577552 |
| May | 0.573050057507233 |
| June 2015 | 0.487560677761359 |
| July | 0.652704468929042 |
| May | 0.828440992383765 |
| June 2016 | 0.839817683883882 |
| July | 0.923801511681964 |
### Chart:
| Category | Barton |
|---|---|
| May | 0.756272212783459 |
| June 2014 | 0.752779914743591 |
| July | 0.804237242416133 |
| May | 0.453542888122897 |
| June 2015 | 0.355545266767683 |
| July | 0.70187259860681 |
| May | 0.707225166091299 |
| June 2016 | 0.630626030447002 |
| July | 0.880658319114917 |
### Chart:
| Category | Pike |
|---|---|
| May | 0.755430875016956 |
| June 2014 | 0.857481715897328 |
| July | 0.93291695038879 |
| May | 0.762960604911385 |
| June 2015 | 0.544089001148871 |
| July | 0.547324428313549 |
| May | 0.915986750540664 |
| June 2016 | 0.932046001039433 |
| July | 0.969033139209825 |
### Chart: Cape Girardeau
| Category | Cape Girardeau |
|---|---|
| May | 0.672265844292923 |
| June 2014 | 0.843455165636624 |
| July | 0.93669167028756 |
| May | 0.729704558726521 |
| June 2015 | 0.752539568216238 |
| July | 0.831474274135911 |
| May | 0.850483054432168 |
| June 2016 | 0.868555848420783 |
| July | 0.969839940187636 |
### Chart:
| Category | Stoddard |
|---|---|
| May | 0.203821207338676 |
| June 2014 | 0.413060707624177 |
| July | 0.61791161807008 |
| May | 0.21011283664305 |
| June 2015 | 0.340049816533958 |
| July | 0.44196571901886 |
| May | 0.418191507658563 |
| June 2016 | 0.465469713269605 |
| July | 0.815326209632442 |
### Chart:
| Category | Cooper |
|---|---|
| May | 0.706205998775803 |
| June 2014 | 0.868226071642684 |
| July | 0.899714110363183 |
| May | 0.716878509230395 |
| June 2015 | 0.653308223346182 |
| July | 0.723998531793833 |
| May | 0.873866664036383 |
| June 2016 | 0.89547692934875 |
| July | 0.928135409376123 |B
A
Probability of A. toschiella biotype 1 occurrence (mean ± SE)
C
D
F
E
Sample Month and Year
S1 Figure. Probability of A. tosichella biotype 1 occurrence (mean ± SE) at locations in Barton (A), Pettis (B), Cape Girardeau (C), Pike (D), Copper (E) and Stoddard (F) county Missouri in 2014, 2015 and 2016.
