# Supplementary material for: Modeling Aceria tosichella biotype distribution over geographic space and time
Source: PLoS One. 2020 May 29;15(5):e0233507. doi: 10.1371/journal.pone.0233507 (PMC7259573; doi:10.1371/journal.pone.0233507)
Supplement: S2 Fig — (PPTX) [file pone.0233507.s002.pptx]

## Slide 1
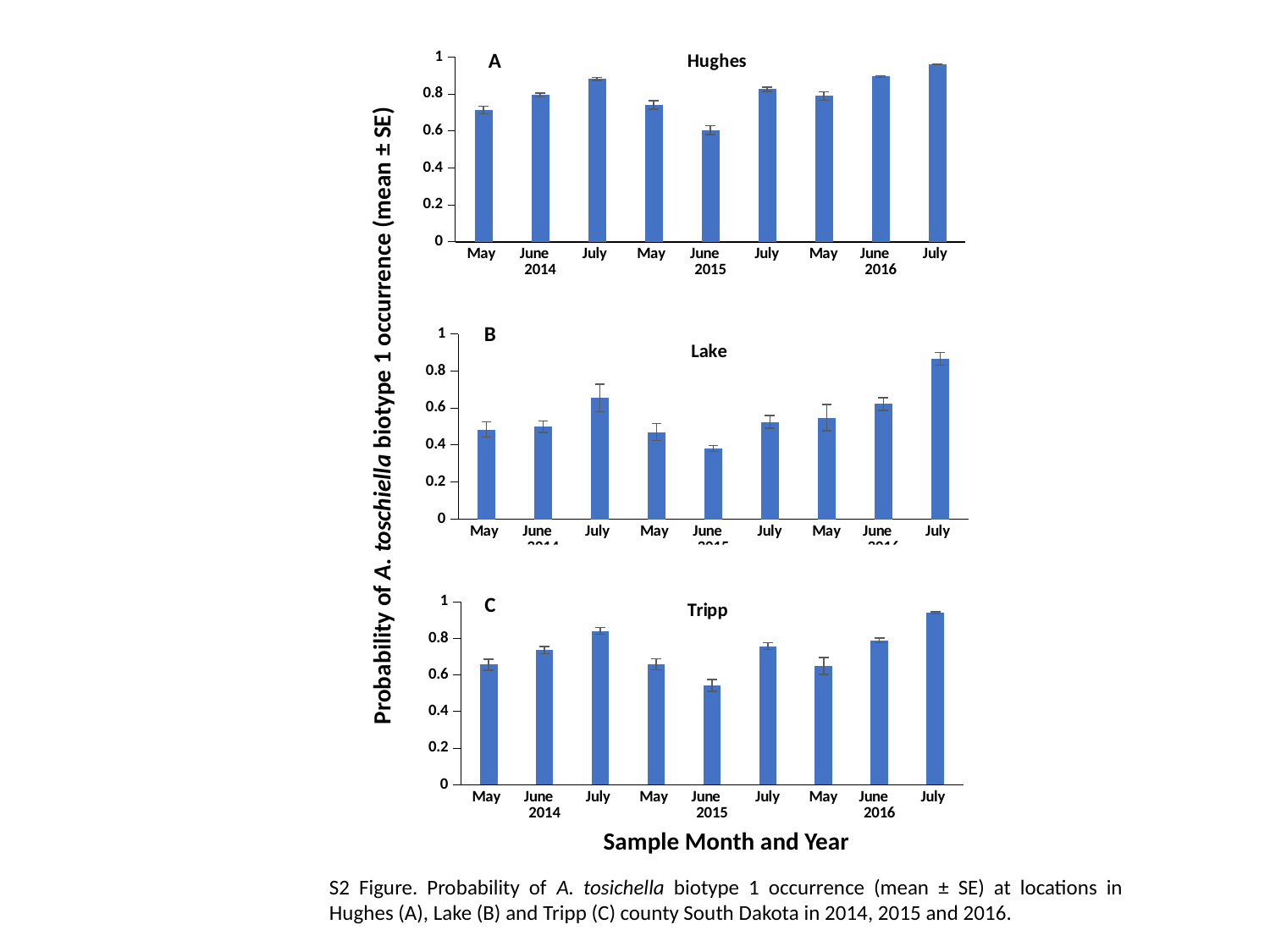

### Chart:
| Category | Hughes |
|---|---|
| May | 0.714177506004412 |
| June 2014 | 0.794865697286804 |
| July | 0.883385607143318 |
| May | 0.741538700935106 |
| June 2015 | 0.605301288997472 |
| July | 0.826843474424946 |
| May | 0.790529647767258 |
| June 2016 | 0.895538246890946 |
| July | 0.962263012299383 |
### Chart:
| Category | Lake |
|---|---|
| May | 0.483373168364237 |
| June 2014 | 0.498809512711182 |
| July | 0.654456799842941 |
| May | 0.470216728562834 |
| June 2015 | 0.380774195564143 |
| July | 0.524833351750617 |
| May | 0.548236228135965 |
| June 2016 | 0.621512761311363 |
| July | 0.865779567005387 |
### Chart:
| Category | Tripp |
|---|---|
| May | 0.656273425072453 |
| June 2014 | 0.735992350360272 |
| July | 0.840919004025089 |
| May | 0.658994603613935 |
| June 2015 | 0.542197404689638 |
| July | 0.757743029952341 |
| May | 0.649418870610205 |
| June 2016 | 0.789308265161162 |
| July | 0.939803143065139 |A
Probability of A. toschiella biotype 1 occurrence (mean ± SE)
B
C
Sample Month and Year
S2 Figure. Probability of A. tosichella biotype 1 occurrence (mean ± SE) at locations in Hughes (A), Lake (B) and Tripp (C) county South Dakota in 2014, 2015 and 2016.
