# Supplementary material for: Modeling Aceria tosichella biotype distribution over geographic space and time
Source: PLoS One. 2020 May 29;15(5):e0233507. doi: 10.1371/journal.pone.0233507 (PMC7259573; doi:10.1371/journal.pone.0233507)
Supplement: S3 Fig — (PPTX) [file pone.0233507.s003.pptx]

## Slide 1
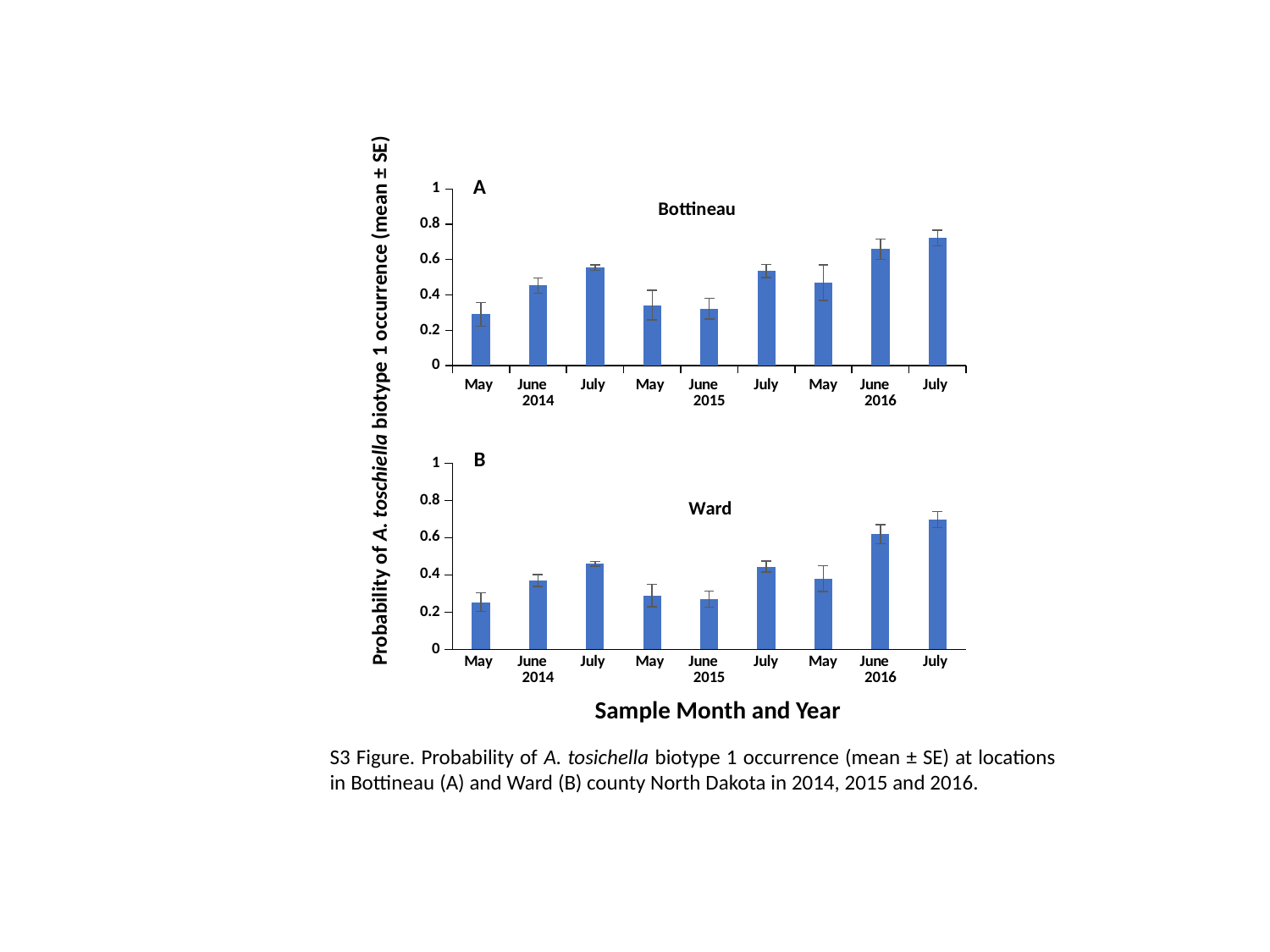

Probability of A. toschiella biotype 1 occurrence (mean ± SE)
### Chart:
| Category | Bottineau |
|---|---|
| May | 0.289955017454197 |
| June 2014 | 0.453007837068744 |
| July | 0.554233131831894 |
| May | 0.341341731902889 |
| June 2015 | 0.322461793478212 |
| July | 0.534475857690651 |
| May | 0.469346139900173 |
| June 2016 | 0.658770626892001 |
| July | 0.72184282983054 |
### Chart:
| Category | Ward |
|---|---|
| May | 0.252918396331345 |
| June 2014 | 0.369175586683493 |
| July | 0.45939680969183 |
| May | 0.289122901301816 |
| June 2015 | 0.269264727359512 |
| July | 0.44330629469672 |
| May | 0.37916948513255 |
| June 2016 | 0.618680033135232 |
| July | 0.697828594826823 |A
B
Sample Month and Year
S3 Figure. Probability of A. tosichella biotype 1 occurrence (mean ± SE) at locations in Bottineau (A) and Ward (B) county North Dakota in 2014, 2015 and 2016.
