# Supplementary material for: Modeling Aceria tosichella biotype distribution over geographic space and time
Source: PLoS One. 2020 May 29;15(5):e0233507. doi: 10.1371/journal.pone.0233507 (PMC7259573; doi:10.1371/journal.pone.0233507)
Supplement: S4 Fig — (PPTX) [file pone.0233507.s004.pptx]

## Slide 1
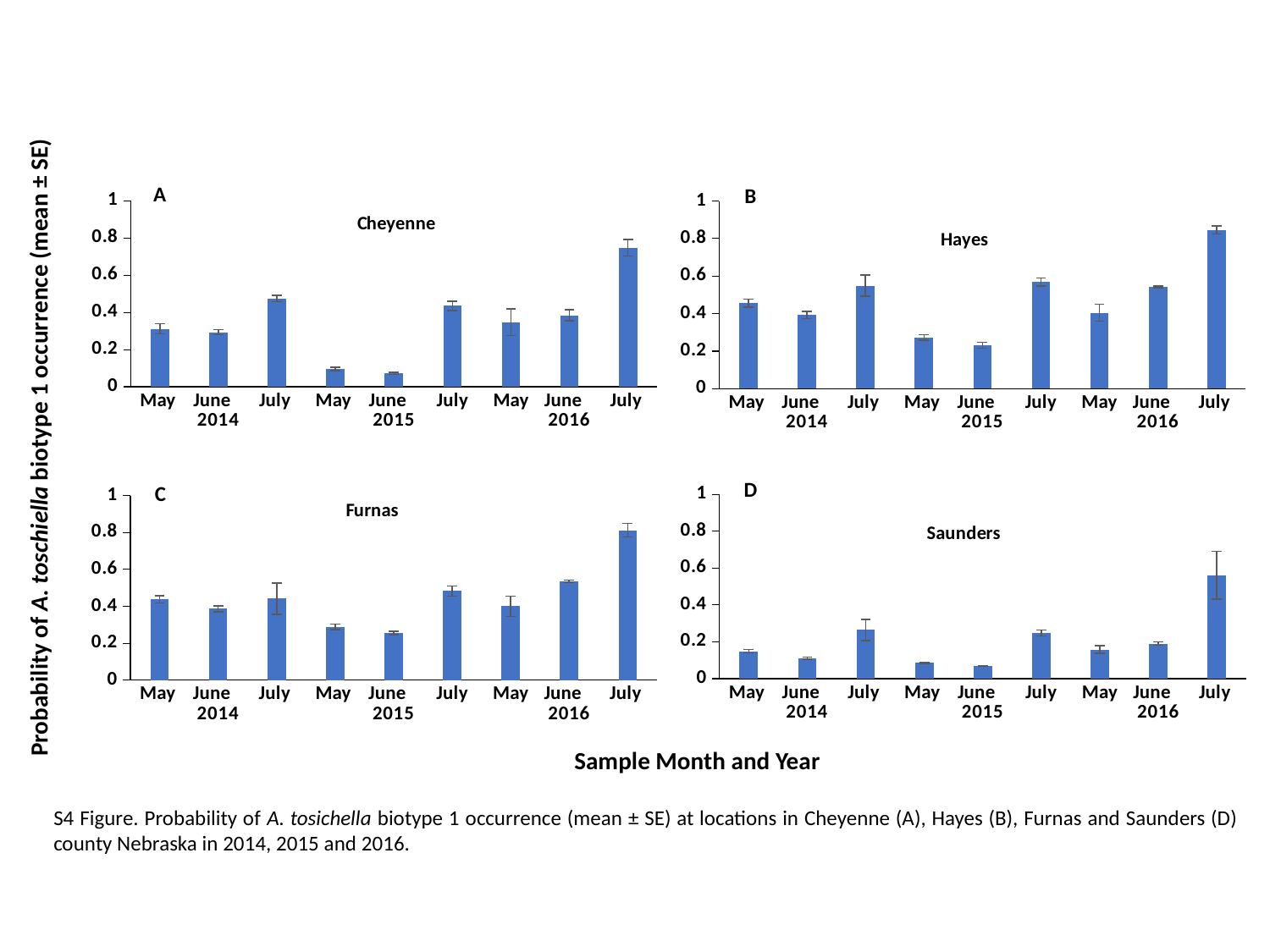

Probability of A. toschiella biotype 1 occurrence (mean ± SE)
### Chart:
| Category | Cheyenne |
|---|---|
| May | 0.311875076238977 |
| June 2014 | 0.293121589183867 |
| July | 0.474626654717618 |
| May | 0.0946133445510161 |
| June 2015 | 0.0712461033961711 |
| July | 0.434816972017118 |
| May | 0.347008537789946 |
| June 2016 | 0.384173210693771 |
| July | 0.747631605340042 |
### Chart:
| Category | Hayes |
|---|---|
| May | 0.45621730800718 |
| June 2014 | 0.39273086399842 |
| July | 0.549379750408104 |
| May | 0.273412786296927 |
| June 2015 | 0.231120839360581 |
| July | 0.56924430663199 |
| May | 0.405066102344198 |
| June 2016 | 0.542040648790927 |
| July | 0.84663820849049 |
### Chart:
| Category | Saunders |
|---|---|
| May | 0.148264016830002 |
| June 2014 | 0.111768005564458 |
| July | 0.264220667369022 |
| May | 0.0861151257125336 |
| June 2015 | 0.0694306977608709 |
| July | 0.247959640522661 |
| May | 0.15764535619669 |
| June 2016 | 0.190056862258976 |
| July | 0.561577623429262 |
### Chart: Furnas
| Category | Furnas |
|---|---|
| May | 0.43718015824858 |
| June 2014 | 0.386915664154422 |
| July | 0.441252381571756 |
| May | 0.288811738219831 |
| June 2015 | 0.255789149038096 |
| July | 0.482725308807093 |
| May | 0.400443992726581 |
| June 2016 | 0.536161336865549 |
| July | 0.812710354488884 |A
B
D
C
Sample Month and Year
S4 Figure. Probability of A. tosichella biotype 1 occurrence (mean ± SE) at locations in Cheyenne (A), Hayes (B), Furnas and Saunders (D) county Nebraska in 2014, 2015 and 2016.
