# Supplementary material for: Modeling Aceria tosichella biotype distribution over geographic space and time
Source: PLoS One. 2020 May 29;15(5):e0233507. doi: 10.1371/journal.pone.0233507 (PMC7259573; doi:10.1371/journal.pone.0233507)
Supplement: S5 Fig — (PPTX) [file pone.0233507.s005.pptx]

## Slide 1
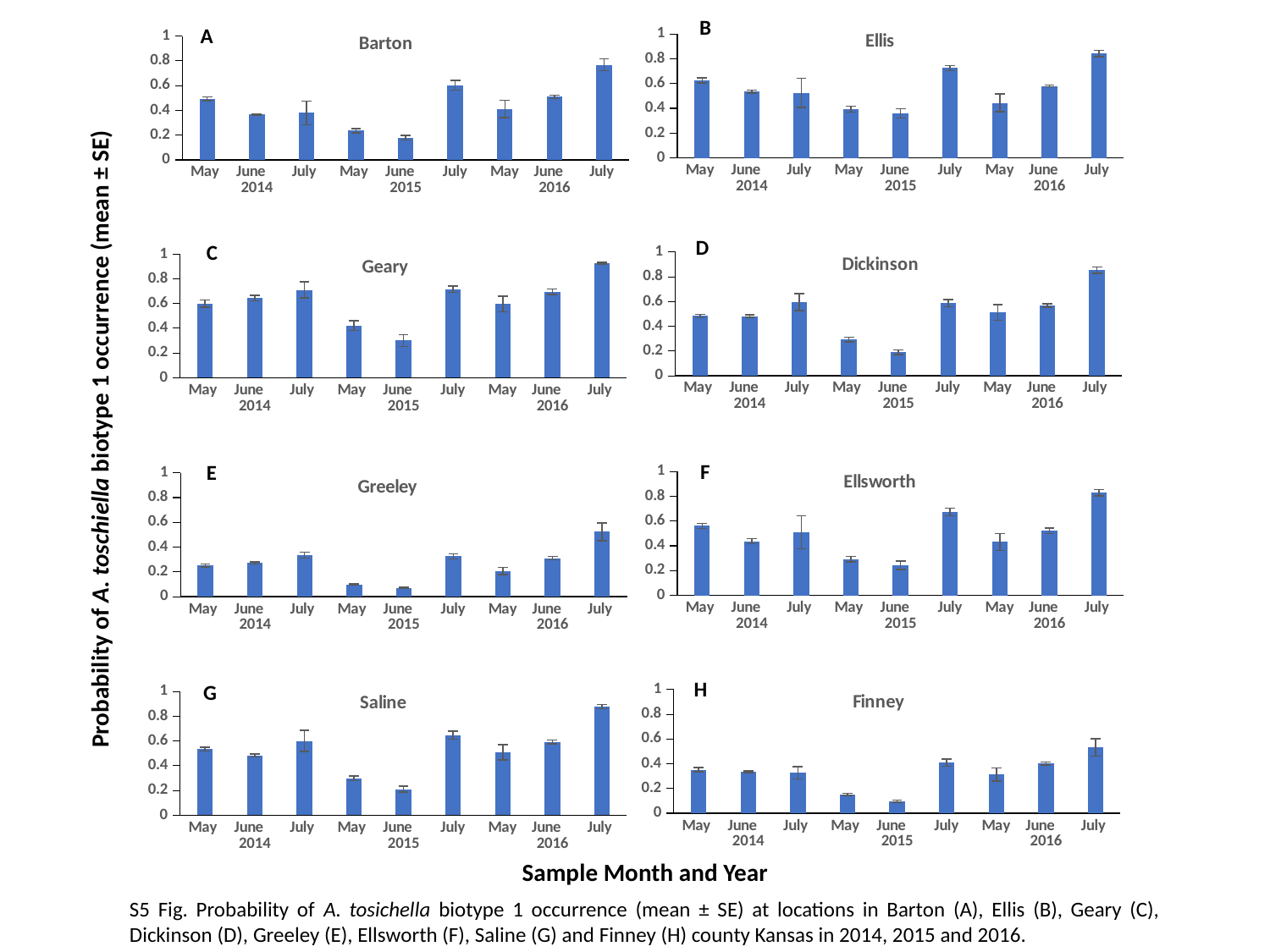

### Chart:
| Category | Ellis |
|---|---|
| May | 0.624812645555518 |
| June 2014 | 0.533996814109334 |
| July | 0.524765672654819 |
| May | 0.393412438178005 |
| June 2015 | 0.3599057388034 |
| July | 0.724212315816161 |
| May | 0.443487783059907 |
| June 2016 | 0.580667431324158 |
| July | 0.842137572718851 |
### Chart:
| Category | Barton |
|---|---|
| May | 0.493512584629526 |
| June 2014 | 0.366261731745187 |
| July | 0.379251314396444 |
| May | 0.235153074693248 |
| June 2015 | 0.179163040085729 |
| July | 0.602880094556468 |
| May | 0.409908932742286 |
| June 2016 | 0.508979956629882 |
| July | 0.767442479860133 |
### Chart:
| Category | Dickinson |
|---|---|
| May | 0.483634494544013 |
| June 2014 | 0.481788819457137 |
| July | 0.59574609894693 |
| May | 0.293458151345779 |
| June 2015 | 0.190475350244618 |
| July | 0.587399962751137 |
| May | 0.511348479689118 |
| June 2016 | 0.56960753193169 |
| July | 0.853915712209909 |
### Chart:
| Category | Geary |
|---|---|
| May | 0.59860705942786 |
| June 2014 | 0.643680774317918 |
| July | 0.709545726907439 |
| May | 0.42209959535454 |
| June 2015 | 0.300304137983268 |
| July | 0.715089627836695 |
| May | 0.597445220309916 |
| June 2016 | 0.695205361861179 |
| July | 0.92651716192427 |
### Chart:
| Category | Ellsworth |
|---|---|
| May | 0.56075902540054 |
| June 2014 | 0.436690739794889 |
| July | 0.508928302524714 |
| May | 0.292069235129407 |
| June 2015 | 0.242329131279026 |
| July | 0.673508938110762 |
| May | 0.431585295223836 |
| June 2016 | 0.521203281066301 |
| July | 0.828401558277907 |
### Chart:
| Category | Greeley |
|---|---|
| May | 0.251191319618754 |
| June 2014 | 0.271965249909389 |
| July | 0.335843923418486 |
| May | 0.099010219028157 |
| June 2015 | 0.071634430537623 |
| July | 0.325425154789636 |
| May | 0.206961820092973 |
| June 2016 | 0.310869044753269 |
| July | 0.523003366941356 |
### Chart:
| Category | Finney |
|---|---|
| May | 0.351616556425108 |
| June 2014 | 0.334498480436739 |
| July | 0.327805769712289 |
| May | 0.151994902226994 |
| June 2015 | 0.0965277661215844 |
| July | 0.408906749312506 |
| May | 0.312814834510608 |
| June 2016 | 0.40129480058988 |
| July | 0.53089382120246 |
### Chart:
| Category | Saline |
|---|---|
| May | 0.534695017002818 |
| June 2014 | 0.482222985627423 |
| July | 0.599541628860467 |
| May | 0.297780931834487 |
| June 2015 | 0.210432229985373 |
| July | 0.646376620001044 |
| May | 0.508122818246952 |
| June 2016 | 0.592982347953923 |
| July | 0.877428401397063 |B
A
Probability of A. toschiella biotype 1 occurrence (mean ± SE)
D
C
F
E
H
G
Sample Month and Year
S5 Fig. Probability of A. tosichella biotype 1 occurrence (mean ± SE) at locations in Barton (A), Ellis (B), Geary (C), Dickinson (D), Greeley (E), Ellsworth (F), Saline (G) and Finney (H) county Kansas in 2014, 2015 and 2016.
