# Supplementary material for: Modeling Aceria tosichella biotype distribution over geographic space and time
Source: PLoS One. 2020 May 29;15(5):e0233507. doi: 10.1371/journal.pone.0233507 (PMC7259573; doi:10.1371/journal.pone.0233507)
Supplement: S6 Fig — (PPTX) [file pone.0233507.s006.pptx]

## Slide 1
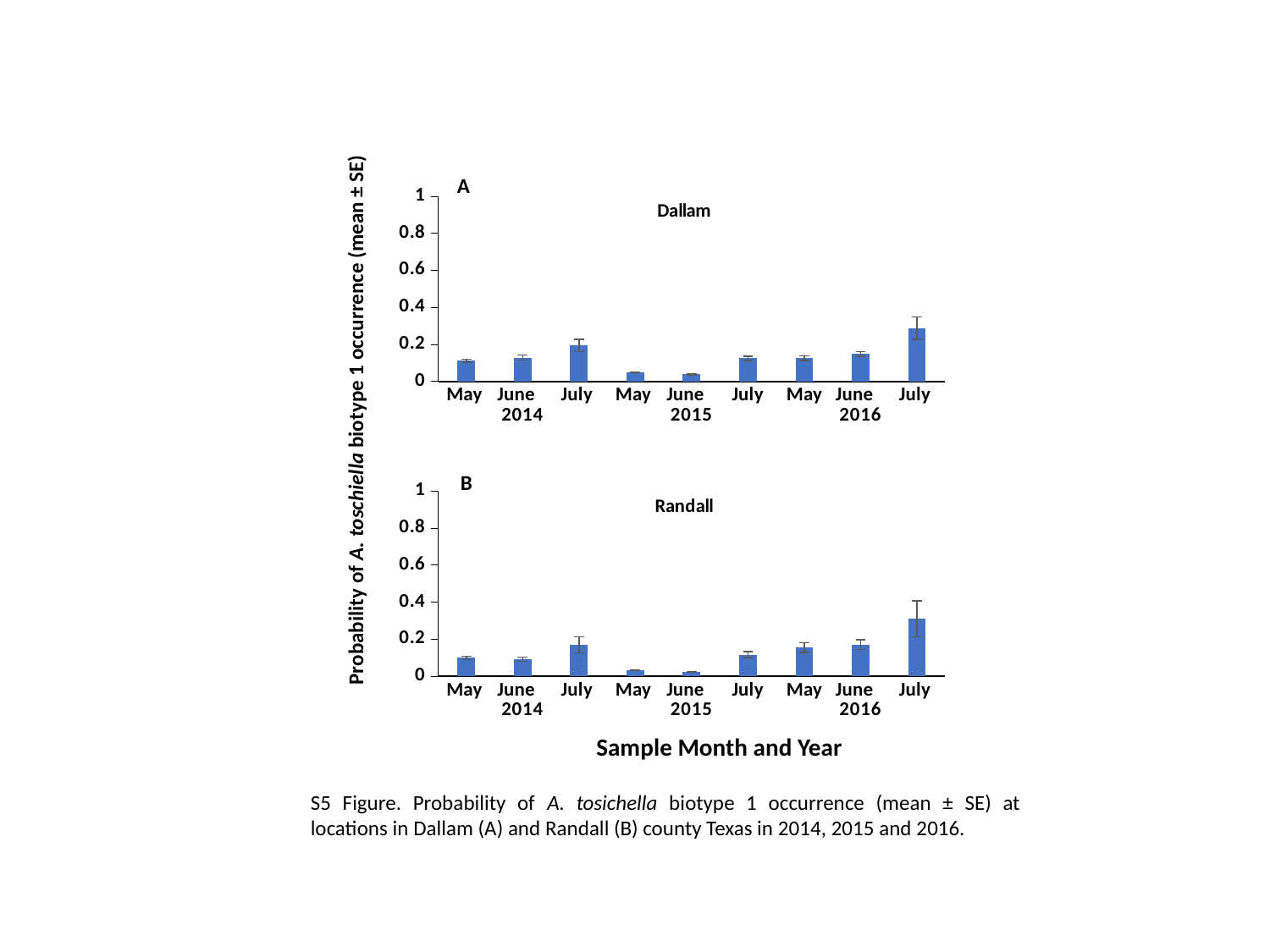

Probability of A. toschiella biotype 1 occurrence (mean ± SE)
### Chart:
| Category | Dallam |
|---|---|
| May | 0.112961560481093 |
| June 2014 | 0.129697735074053 |
| July | 0.195794137643528 |
| May | 0.0499438475261658 |
| June 2015 | 0.0395028637823446 |
| July | 0.125807653787223 |
| May | 0.12632074088996 |
| June 2016 | 0.148634929754245 |
| July | 0.288486770476886 |
### Chart:
| Category | Randall |
|---|---|
| May | 0.0995660505490792 |
| June 2014 | 0.0915949776235828 |
| July | 0.16932511552107 |
| May | 0.0314320517012577 |
| June 2015 | 0.021513404784998 |
| July | 0.116367454922577 |
| May | 0.155565554028542 |
| June 2016 | 0.171069587205107 |
| July | 0.310001420504698 |A
B
Sample Month and Year
S5 Figure. Probability of A. tosichella biotype 1 occurrence (mean ± SE) at locations in Dallam (A) and Randall (B) county Texas in 2014, 2015 and 2016.
