# Supplementary material for: Modeling Aceria tosichella biotype distribution over geographic space and time
Source: PLoS One. 2020 May 29;15(5):e0233507. doi: 10.1371/journal.pone.0233507 (PMC7259573; doi:10.1371/journal.pone.0233507)
Supplement: S1 Table — (DOCX) [file pone.0233507.s007.docx]

S1 Table. State, county, and geographic coordinates for locations of *A. tosichella* samples collected in 2014 and 2015.

| Location | | Geographic coordinate (latitude, longitude)* | | | |
| --- | --- | --- | --- | --- | --- |
| State | County | Field 1 | Field 2 | Field 3 | Sample date (mm.dd.yyyy) |
| Kansas | Saline | 38.8622, -97.5715 | 38.8053, -97.7429 | 38.9061, -97.6477 | 05.21.2014 |
|  | Geary | 39.0447, -96.9122 | 39.0587, -96.8585 | 38.9138, -96.6132 | 05.21.2014 |
|  | Finney | 38.0591, -100.3221 | 38.0590, -100.4634 | 38.0590, -100.4634 | 06.04.2014 |
|  | Dickinson | 38.6963, -97.2344 | 39.0385, -97.2163 | 39.0312, -97.2351 | 06.04.2014 |
|  | Ellsworth | 38.7306, -98.2999 | 38.5659, -98.4440 | 38.8128, -98.2433 | 06.04.2014 |
|  | Greeley | 38.2915, -101.7530 | 38.3643, -101.7530 | 38.4368, -101.7338 | 06.04.2014 |
|  | Barton | 38.4845, -98.5546 | 38.3277, -98.8479 | 38.3277,-98.8479 | 06.04.2014 |
|  | Ellis | 39.0449, -99.3167 | 38.9144, -99.3251 | 38.7405, -99.3176 | 06.04.2014 |
| South Dakota | Hughes | 44.5199, -100.4512 | 44.5187, -99.7029 | 44.5174, -99.9466 | 06.20.2014 |
|  | Lake | 44.0660, -96.9491 | 44.0586, -97.0895 | 43.8998, -97.0693 | 06.25.2015 |
|  | Tripp | 43.4298, -99.8500 | 43.4174, -99.8506 | 43.4153, -99.8435 | 06.28.2015 |
| North Dakota | Ward | 48.4441, -101.1904 | 48.4256, -101.1470 | 48.2694, -101.7068 | 07.08.2014 |
|  | Bottineau | 48.6900, -100.3413 | 48.7666, -101.0703 | 48.7630, -101.0703 | 07.08.2014 |
| Nebraska | Cheyenne | 41.3516, -102.7186 | 41.3080, -102.9381 | 41.3079, -102.9348 | 07.10.2014 |
|  | Hayes | 40.4251, -101.0986 | 40.6475, -101.0463 | 40.6450, -101.0623 | 05.10.2015 |
|  | Furnas | 40.0446, -100.1307 | 40.0093, -99.8934 | 40.1416, -99.8948 | 06.10.2015 |
|  | Saunders | 41.3573, -96.5603 | 41.3575, -96.6660 | 41.3583, -96.6594 | 07.10.2015 |
| Missouri | Barton | 37.6177, -94.2938 | 37.6180, -94.3021 | 37.3982, -94.2887 | 07.12.2015 |
|  | Cape Girardeau | 37.5424, -89.6553 | 37.5320, -89.6751 | 37.5743, -89.7299 | 07.12.2015 |
|  | Pike | 39.3282, -90.9945 | 39.3295, -91.1364 | 39.2038, -91.3620 | 07.12.2015 |
|  | Pettis | 38.7018, -93.1058 | 38.7052, -93.4210 | 38.7038, -93.4126 | 07.12.2015 |
|  | Stoddard | 36.9748, -89.7614 | 36.9552, -90.0749 | 36.9582, -90.0748 | 07.12.2015 |
|  | Cooper | 38.7890, -92.6514 | 38.8029, -92.8940 | 38.8029, -92.8939 | 07.12.2015 |
| Texas | Randall | - | - | - | - |
|  | Dallam | - | - | - | - |

- Recorded using a hand-held GPS device
