# Supplementary material for: Modeling Aceria tosichella biotype distribution over geographic space and time
Source: PLoS One. 2020 May 29;15(5):e0233507. doi: 10.1371/journal.pone.0233507 (PMC7259573; doi:10.1371/journal.pone.0233507)
Supplement: S2 Table — (DOCX) [file pone.0233507.s008.docx]

S2 Table. State, county, and geographic coordinates for locations of *A. tosichella* samples collected in 2016.

| Location | | | | | Geographic Coordinate (latitude, longitude)* | | | | | | |
| --- | --- | --- | --- | --- | --- | --- | --- | --- | --- | --- | --- |
| State | County | Field site | | Field 1 | | | Field 2 | | Field 3 | Collection date (mm.dd.yyyy) |  |
| Missouri | Barton | | 1 | | | 37.7352, -94.4532 | | 37.6178, -94.2940 | 37.3983, -94.2949 | 06.11.2016 |  |
|  |  | | 2 | | | 37.7352, -94.4532 | | 37.6178, -94.2958 | 37.3983, -94.2864 | 06.11.2016 |  |
|  |  | | 3 | | | 37.7412, -94.4527 | | 37.6178, -94.2948 | 37.3994, -94.2861 | 06.11.2016 |  |
|  | Cape Girardeau | | 1 | | | 37.5420, -89.6559 | | 37.5254, -89.6701 | 37.3993, -89.2861 | 06.12.2016 |  |
|  |  | | 2 | | | 37.5414, -89.6579 | | 37.5265, -89.6701 | 37.6010, -89.7289 | 06.12.2016 |  |
|  |  | | 3 | | | 37.5426, -89.6550 | | 37.5272, -89.6701 | 37.6022, -89.7291 | 06.12.2016 |  |
| Kansas | Ellis | | 1 | | | 38.8992, -99.5546 | | 38.8991, -99.5286 | 38.9082, -99.5210 | 06.16.2016 |  |
|  |  | | 2 | | | 38.8989, -99.5490 | | 38.8994, -99.5257 | 38.9069, -99.5210 | 06.16.2016 |  |
|  |  | | 3 | | | 38.8992, -99.5502 | | 38.8995, -99.5216 | 38.9092, -99.5210 | 06.16.2016 |  |
| Nebraska | Hayes | | 1 | | | 40.6365, -101.0449 | | 40.5346, -101.0274 | 40.4155, -101.0305 | 06.16.2016 |  |
|  |  | | 2 | | | 40.6340, -101.0449 | | 40.5346, -101.0255 | 40.4156, -101.0246 | 06.16.2016 |  |
|  |  | | 3 | | | 40.6387, -101.0450 | | 40.5310, -101.0250 | 40.4156, -101.0273 | 06.16.2016 |  |

- Recorded using a hand-held GPS device
