# Supplementary material for: Modeling Aceria tosichella biotype distribution over geographic space and time
Source: PLoS One. 2020 May 29;15(5):e0233507. doi: 10.1371/journal.pone.0233507 (PMC7259573; doi:10.1371/journal.pone.0233507)
Supplement: S3 Table — (DOCX) [file pone.0233507.s009.docx]

S3 Table. Estimates of genetic distance and genetic identity in eight U.S. *A. tosichella* populations collected in 2014, 2015 and 2016, using variation among unique ITS1 haplotypes.

| Population/Location | 8-3-1 | 8-4-1 | 17-1-1 | 17-10-1 | 18-1-1 | 5-1-1 | 7-1-1 | 27-4-1 |
| --- | --- | --- | --- | --- | --- | --- | --- | --- |
| BT1_8-3-1/ Ellis, KS | - |  |  |  |  |  |  |  |
| BT1_8-4-1/ Ellis, KS | 0.005 | - |  |  |  |  |  |  |
| BT1_17-1-1/ Barton, MO | 0.008 | 0.003 | - |  |  |  |  |  |
| BT1_17-10-1 Barton, MO | 0.016 | 0.013 | 0.015 | - |  |  |  |  |
| BT1_18-1-1/ Cape Girardeau, MO | 0.008 | 0.003 | 0.006 | 0.016 | - |  |  |  |
| BT2_5-1-1/ Dickinson, KS | 0.010 | 0.008 | 0.008 | 0.020 | 0.011 | - |  |  |
| BT2_7-1-1/ Finney, KS | 0.011 | 0.010 | 0.013 | 0.023 | 0.013 | 0.002 | - |  |
| BT2_27-4-1/ Texas | 0.016 | 0.015 | 0.018 | 0.028 | 0.018 | 0.007 | 0.018 | - |
