# Supplementary material for: Modeling Aceria tosichella biotype distribution over geographic space and time
Source: PLoS One. 2020 May 29;15(5):e0233507. doi: 10.1371/journal.pone.0233507 (PMC7259573; doi:10.1371/journal.pone.0233507)
Supplement: S4 Table — (DOCX) [file pone.0233507.s010.docx]

S4 Table. Estimates of genetic distance three U.S. *A. tosichella* populations collected in 2014, 2015 and 2016, using variation among unique COI haplotypes.

| Location/Year/Field | 1 | 2 | 3 |
| --- | --- | --- | --- |
| 1-Barton Co. KS/2014/ Field G | - |  |  |
| 2-Cooper Co. MO/2015/ Field D | 0.174 | - |  |
| 3- Hayes Co. NE/2016/ Field 3.1A | 0.002 | 0.177 | - |
