# Supplementary material for: Modeling Aceria tosichella biotype distribution over geographic space and time
Source: PLoS One. 2020 May 29;15(5):e0233507. doi: 10.1371/journal.pone.0233507 (PMC7259573; doi:10.1371/journal.pone.0233507)
Supplement: S1 Analysis — (PDF) [file pone.0233507.s011.pdf]

Reproducible Analysis

Contents

1 Introduction **2**
- 1.1 Load data . . . 2
- 1.2 Data preparation . . . 2

2 Data analysis **4**
- 2.1 Collinearity . . . 4
- 2.2 Model fitting . . . 4
- 2.3 Model selection . . . 5
- 2.4 Model checking . . . 5
- 2.5 Statistical inference . . . 6
- 2.6 Figure 4 . . . 6
- 2.7 County-level estimates . . . 7

1 Introduction

This appendix provides documentation and R code associated with the analysis presented in the manuscript. The R code in this appendix was designed to be executed in the order it is presented due to dependence on prior code. We begin by loading the required R packages.

```
library(mgcv)
library(lubridate)
library(raster)
library(rasterVis)
library(prism)
library(fields)
library(msm)
require(plotrix)
library(maps)
library(maptools)
```

1.1 Load data

Next we need to load the data. After publication all of the data required to reproduce the results of the spatio-temporal statistical analysis will be available on the Dryad Digital repository (Khalaf et al. 2020). For the purpose of peer review, the data have been submitted as supporting material as a compressed file labeled Data.zip. Once Data.zip is downloaded and uncompressed, set your R working directory to the main folder (i.e., the folder "Data"). The R code below takes approximately 10 minutes to run.

```
# National Land Cover Database
file.nlcd <- "NLCD/StudyArea_2011_NLCD.img"
rl.nlcd2011 <- raster(file.nlcd)
sf.studyarea <- as(extent(rl.nlcd2011), "SpatialPolygons")
proj4string(sf.studyarea) <- crs(rl.nlcd2011)
rl.grass <- rl.nlcd2011 == 71
rl.pasture <- rl.nlcd2011 == 81
rl.grass.pasture <- rl.pasture + rl.grass
rl.crop <- rl.nlcd2011 == 82

# State Maps
map.usa <- map("state", fill = TRUE, plot = FALSE)
map.crs <- CRS("+proj=longlat +datum=WGS84 +no_defs +ellps=WGS84 +towgs84=0,0,0")
sf.usa <- map2SpatialPolygons(map.usa, IDs = map.usa$names, proj4string = map.crs)
sf.usa <- spTransform(sf.usa, CRS(projection(rl.nlcd2011)))

# PRISM (Parameter-elevation Regressions on Independent Slopes Model) Data
options(prism.path = "PRISM")

# Download directly from PRISM get_prism_monthlys(type='ppt', years = 2014:2016, mon =
# 1:12, keepZip=FALSE) get_prism_monthlys(type='tmean', years = 2014:2016, mon = 1:12,
# keepZip=FALSE)

# Load from local hardrive
rs.precip <- prism_stack(ls_prism_data()[1:36, ])
rs.precip <- crop(rs.precip, spTransform(buffer(sf.studyarea, 20000), crs(rs.precip)))
rs.tmean <- prism_stack(ls_prism_data()[37:72, ])
rs.tmean <- crop(rs.tmean, spTransform(buffer(sf.studyarea, 20000), crs(rs.tmean)))

# Wheat Curl Mite Data (Original data set)
file.wcm.orig <- "Wheat Curl Mite Data/wcm data.csv"
df.wcm.orig <- read.csv(file.wcm.orig)
```

1.2 Data preparation

The original wheat curl mite data set contains the number of individuals of biotype 1 and 2 at a sampled date and location (latitude and longitude), which is contained within the data frame `df.wcm.orig`. In addition to spatial and temporal dynamics, the statistical models used in our study can incorporate covariates (i.e., predictor variables). The code below adds the covariates to the data frame `df.wcm.orig`, which is used to produce the data frame `df.wcm`.

```
# Wheat Curl Mite Data Prep
df.wcm.orig$Collection.date <- as.Date(df.wcm.orig$Collection.date, "%m.%d.%Y")
pts.sample <- df.wcm.orig
coordinates(pts.sample) = ~long + lat
proj4string(pts.sample) <- CRS("+proj=longlat +datum=WGS84 +no_defs +ellps=WGS84 +towgs84=0,0,0")
pts.sample <- spTransform(pts.sample, CRS(projection(rl.nlcd2011)))

df.wcm <- data.frame(pts.sample)[, -7]
df.wcm$month <- month(df.wcm$Collection.date) + ifelse(year(df.wcm$Collection.date) ==
    2014, 0, ifelse(year(df.wcm$Collection.date) == 2015, 12, 24))  #Months since Jan 1 2014
df.wcm$year <- year(df.wcm$Collection.date)

df.wcm$perc.grass.pasture.100 <- unlist(lapply(extract(rl.grass.pasture, pts.sample,
    buffer = 100), mean))
df.wcm$perc.crop.100 <- unlist(lapply(extract(rl.crop, pts.sample, buffer = 100),
    mean))

df.wcm$perc.grass.pasture.500 <- unlist(lapply(extract(rl.grass.pasture, pts.sample,
    buffer = 500), mean))
df.wcm$perc.crop.500 <- unlist(lapply(extract(rl.crop, pts.sample, buffer = 500),
    mean))

df.wcm$perc.grass.pasture.1000 <- unlist(lapply(extract(rl.grass.pasture, pts.sample,
    buffer = 1000), mean))
df.wcm$perc.crop.1000 <- unlist(lapply(extract(rl.crop, pts.sample, buffer = 1000),
    mean))

df.wcm$perc.grass.pasture.2500 <- unlist(lapply(extract(rl.grass.pasture, pts.sample,
    buffer = 2500), mean))
df.wcm$perc.crop.2500 <- unlist(lapply(extract(rl.crop, pts.sample, buffer = 2500),
    mean))

df.wcm$perc.grass.pasture.5000 <- unlist(lapply(extract(rl.grass.pasture, pts.sample,
    buffer = 5000), mean))
df.wcm$perc.crop.5000 <- unlist(lapply(extract(rl.crop, pts.sample, buffer = 5000),
    mean))

# Required functions
get.temporal.x <- function(raster, points, t, max.lag) {
    X <- extract(raster, points)
    n <- dim(X)[1]
    T <- dim(X)[2]
    D <- rdist(t, 1:T)
    for (i in 1:dim(D)[1]) {
        D[i, (which.min(D[i, ]) + 1):T] <- Inf
    }
    X.lag <- matrix(, n, max.lag + 1)
    for (i in 1:(max.lag + 1)) {
        keep <- apply(D, 1, function(x) {
            which(x == (i - 1))
        })
        X.lag[, i] <- X[cbind(1:n, keep)]
    }
    X.lag
}

points <- spTransform(pts.sample, CRS(projection(rs.precip)))
x.precip <- get.temporal.x(rs.precip, points, df.wcm$month, 1)
colnames(x.precip) <- c("precip.1", "precip.2")
x.tmean <- get.temporal.x(rs.tmean, points, df.wcm$month, 1)
colnames(x.tmean) <- c("tmean.1", "tmean.2")
df.wcm <- cbind(df.wcm, x.precip, x.tmean)
```

2 Data analysis

2.1 Collinearity

Climate and land cover variables that are used as covariates are often correlated. When using regression-type models, correlation among the covariates results in the well-known problem of collinearity (Dormann et al. 2013). Collinear covariates have the potential to make regression coefficient estimates highly variable (i.e., have wide confidence intervals) and may also result in models where inference is sensitive to small changes in model specification (e.g., the inclusions or exclusions of a single covariate may influence the inference for other covariates in a model). Below we calculate the coefficient of determination (R^2) for pairs of the covariates.

```
cor(df.wcm[, 9:10])  # Correlation among land cover covariates (100 m)

                       perc.grass.pasture.100 perc.crop.100
perc.grass.pasture.100               1.000000     -0.717481
perc.crop.100                       -0.717481      1.000000

cor(df.wcm[, 11:12])  # Correlation among land cover covariates (500 m)

                       perc.grass.pasture.500 perc.crop.500
perc.grass.pasture.500              1.0000000    -0.8895612
perc.crop.500                      -0.8895612     1.0000000

cor(df.wcm[, 13:14])  # Correlation among land cover covariates (1000 m)

                        perc.grass.pasture.1000 perc.crop.1000
perc.grass.pasture.1000               1.0000000     -0.8728023
perc.crop.1000                       -0.8728023      1.0000000

cor(df.wcm[, 15:16])  # Correlation among land cover covariates (2500 m)

                        perc.grass.pasture.2500 perc.crop.2500
perc.grass.pasture.2500               1.0000000     -0.7916841
perc.crop.2500                       -0.7916841      1.0000000

cor(df.wcm[, 17:18])  # Correlation among land cover covariates (5000 m)

                        perc.grass.pasture.5000 perc.crop.5000
perc.grass.pasture.5000               1.0000000     -0.7824858
perc.crop.5000                       -0.7824858      1.0000000

cor(df.wcm[, 19:22])  # Correlation among climate covariates

            precip.1   precip.2   tmean.1   tmean.2
precip.1  1.0000000 -0.0709517 0.1421885 0.4656008
precip.2 -0.0709517  1.0000000 0.4264979 0.4158015
tmean.1   0.1421885  0.4264979 1.0000000 0.7081616
tmean.2   0.4656008  0.4158015 0.7081616 1.0000000
```

2.2 Model fitting

Below is the R code that fits the generalized additive models described in the manuscript.

```
m.null <- gam(cbind(df.wcm$Type.1, df.wcm$Type.2) ~ 1, family = binomial, data = df.wcm)

m.100 <- bam(cbind(df.wcm$Type.1, df.wcm$Type.2) ~ s(lat, long, bs = "tp", k = 10) +
    factor(year) + perc.grass.pasture.100 * perc.crop.100 + precip.1 + precip.2 +
    tmean.1 + tmean.2, family = binomial, data = df.wcm)

m.500 <- bam(cbind(df.wcm$Type.1, df.wcm$Type.2) ~ s(lat, long, bs = "tp", k = 10) +
    factor(year) + perc.grass.pasture.500 * perc.crop.500 + precip.1 + precip.2 +
    tmean.1 + tmean.2, family = binomial, data = df.wcm)

m.1000 <- bam(cbind(df.wcm$Type.1, df.wcm$Type.2) ~ s(lat, long, bs = "tp",
    k = 10) + factor(year) + perc.grass.pasture.1000 * perc.crop.1000 + precip.1 +
```

```
    precip.2 + tmean.1 + tmean.2, family = binomial, data = df.wcm)

m.2500 <- bam(cbind(df.wcm$Type.1, df.wcm$Type.2) ~ s(lat, long, bs = "tp",
    k = 10) + factor(year) + perc.grass.pasture.2500 * perc.crop.2500 + precip.1 +
    precip.2 + tmean.1 + tmean.2, family = binomial, data = df.wcm)

m.5000 <- bam(cbind(df.wcm$Type.1, df.wcm$Type.2) ~ s(lat, long, bs = "tp",
    k = 10) + factor(year) + perc.grass.pasture.5000 * perc.crop.5000 + precip.1 +
    precip.2 + tmean.1 + tmean.2, family = binomial, data = df.wcm)
```

2.3 Model selection

As described in the manuscript, we used Akaike information criterion (AIC) to compare models and determine the appropriate scale for the land cover covariates. The code below gives the AIC scores for each scale. We chose the scale with the lowest AIC score, which was 5000m.

```
AIC(m.null, m.100, m.500, m.1000, m.2500, m.5000)

             df      AIC
m.null  1.00000 219.1174
m.100  18.67261 172.0321
m.500  18.64217 173.7147
m.1000 18.75003 169.8558
m.2500 11.99980 171.9495
m.5000 17.93751 162.6826
```

We also report the "deviance explained," which is a measure of predictive accuracy that is similar to the coefficient of determination (R^2), but is more appropriate for our models (Wood 2017).

```
summary(m.null)$dev

[1] -4.468926e-16

summary(m.100)$dev

[1] 0.6480531

summary(m.500)$dev

[1] 0.6343464

summary(m.1000)$dev

[1] 0.6663799

summary(m.2500)$dev

[1] 0.5437816

summary(m.5000)$dev

[1] 0.7099987
```

2.4 Model checking

As described in the manuscript, we preformed a variety of diagnostics to check the assumptions of our top model (i.e., the model with the lowest AIC score). Similar model diagnostics were preformed by Wood (2017; pg. 356) and we refer to reader to this source for more detail.

```
par(mfrow = c(2, 4))
plot(df.wcm$perc.grass.pasture.5000, residuals(m.5000))
plot(df.wcm$perc.crop.5000, residuals(m.5000))
plot(df.wcm$precip.1, residuals(m.5000))
```

```
plot(df.wcm$precip.2, residuals(m.5000))
plot(df.wcm$tmean.1, residuals(m.5000))
plot(df.wcm$tmean.2, residuals(m.5000))
qq.gam(m.5000, rep = 1000)
```

2.5 Statistical inference

After selecting the model with the appropriate scale for the land cover covariates, we calculated point estimates and 90% confidence interval for the regression coefficients associated with the climate and land cover covariates.

```
results <- cbind(coef(m.5000)[4:10], confint.default(m.5000, level = 0.9)[4:10, ])
colnames(results) <- c("coefficient estimate", "lower limit", "upper limit")
results

                                       coefficient estimate  lower limit
perc.grass.pasture.5000                        -5.407449843 -10.77036684
perc.crop.5000                                 -7.218225534 -11.20881976
precip.1                                       -0.004097512  -0.00865371
precip.2                                       -0.006526856  -0.01333780
tmean.1                                        -0.050180035  -0.28666305
tmean.2                                         0.140444926  -0.08242021
perc.grass.pasture.5000:perc.crop.5000          6.072090929  -2.15890794
                                         upper limit
perc.grass.pasture.5000                -0.0445328466
perc.crop.5000                         -3.2276313088
precip.1                                0.0004586858
precip.2                                0.0002840931
tmean.1                                 0.1863029754
tmean.2                                 0.3633100580
perc.grass.pasture.5000:perc.crop.5000 14.3030898016
```

2.6 Figure 4

The R code below obtains predictions from the generalized additive model using the climate and land cover covariates at the 5000m scale, which is used to produce figure 4 in the manuscript. This R code takes approximately 30 minutes to run. The long run time is a result of the 5000m scale, which requires modification of the land cover raster files.

```
temp1 <- aggregate(rl.grass.pasture, fact = 10000/30)
temp2 <- aggregate(rl.crop, fact = 10000/30)

pts.pred <- rasterToPoints(temp1, spatial = TRUE)
df.pred <- data.frame(pts.pred)[, 2:3]
names(df.pred) <- c("long", "lat")

pts.pred.prism <- spTransform(pts.pred, CRS(projection(rs.precip)))

df.pred$perc.grass.pasture.5000 <- extract(temp1, pts.pred)
df.pred$perc.crop.5000 <- extract(temp2, pts.pred)


rl.pred <- raster(, ext = extent(temp1), resolution = res(temp1))
temp3 <- df.pred
dates <- c(as.Date("2014-05-15"), as.Date("2014-06-15"), as.Date("2014-07-15"),
    as.Date("2015-05-15"), as.Date("2015-06-15"), as.Date("2015-07-15"),
    as.Date("2016-05-15"), as.Date("2016-06-15"), as.Date("2016-07-15"))


for (t in 1:length(dates)) {
    temp4 <- data.frame(lat = temp3[, 2], long = temp3[, 1],
        perc.grass.pasture.5000 = temp3[, 3], perc.crop.5000 = temp3[,
            4], year = year(dates[t]), yday = yday(dates[t]))
    temp4$month <- month(dates[t]) + ifelse(year(dates[t]) ==
```

```
        2014, 0, ifelse(year(dates[t]) == 2015, 12, 24))
    x.precip <- get.temporal.x(rs.precip, pts.pred.prism, temp4$month,
        1)
    temp4$precip.1 <- x.precip[, 1]
    temp4$precip.2 <- x.precip[, 2]
    x.tmean <- get.temporal.x(rs.tmean, pts.pred.prism, temp4$month,
        1)
    temp4$tmean.1 <- x.tmean[, 1]
    temp4$tmean.2 <- x.tmean[, 2]

    temp4[is.na(temp4$tmean.1), 8:11] <- -1
    rl.pred[] <- c(predict(m.5000, type = "response", newdata = temp4))
    rl.pred[which(temp4$tmean.1 == -1)] <- 0

    if (t == 1) {
        rl.pred.all <- rl.pred
    } else {
        rl.pred.all <- stack(rl.pred, rl.pred.all)
    }
}

crs(rl.pred.all) <- crs(sf.usa)
titles <- c("May 2014", "June 2014", "July 2014", "May 2015",
    "June 2015", "July 2015", "May 2016", "June 2016", "July 2016")
names(rl.pred.all) <- titles

rl.pred.all <- mask(rl.pred.all, sf.usa)

# pdf(file='fig_4.pdf',width=7,height = 9)
levelplot(rl.pred.all, cuts = 254, margin = FALSE, names.attr = titles,
    xlab = NULL, ylab = NULL, scales = list(draw = FALSE), par.settings = list(layout.widths = list(right.padding = 6)),
    colorkey = list(space = "right", width = 3, length = 0.5,
        height = 0.9), col.regions = colorRampPalette(rev(brewer.pal(11,
        "Spectral")), bias = 1)) + layer(sp.polygons(sf.usa))
grid.text("Probability of biotype 1", x = 0.99, y = 0.5, just = c("center",
    "bottom"), gp = gpar(cex = 2.1), rot = 90)
# dev.off()
```

2.7 County-level estimates

Using the the generalized additive model with climate and land cover covariates at the 5000m scale, we obtained county-level estimates and associated standard errors of the probability that biotype 1 occurs at any location within the counties of interest. This requires that we take estimates at the point-level in continuous space and upscale to the county-level. Within the field of spatio-temporal statistics, this technique is known as the change-of-support (see Gotway and Young 2002 or Cressie and Wikle 2011 for more details). Briefly, the change-of support involves taking point-level estimates of the probability of biotype 1, $p(\mathbf{s}, t)$, where $\mathbf{s}$ is a vector that contains the coordinates (at a point) and t is the month and scaling this probability up to the county-level. The upscaling is accomplished by calculating $|\mathcal{A}|^{-1} \int_{\mathcal{A}} p(\mathbf{s}, t) d\mathbf{s}$ where $\mathcal{A}$ is the county of interest. The R code below approximates the integral as well as provides standard errors for the estimates.

```
sf.counties <- getData("GADM", country = "USA", level = 2)
sf.counties <- spTransform(sf.counties, CRS(projection(rl.pred.all)))
pts.sample <- df.wcm[, 5:6]
coordinates(pts.sample) = ~long + lat
proj4string(pts.sample) <- CRS(projection(rl.pred.all))
sf.counties <- sf.counties[!is.na(over(sf.counties, pts.sample)), ]

dates <- c(as.Date("2014-05-15"), as.Date("2014-06-15"), as.Date("2014-07-15"), as.Date("2015-05-15"),
    as.Date("2015-06-15"), as.Date("2015-07-15"), as.Date("2016-05-15"), as.Date("2016-06-15"),
    as.Date("2016-07-15"))

df.county.prob <- matrix(, dim(sf.counties)[1], length(dates))
colnames(df.county.prob) <- as.character(dates)
rownames(df.county.prob) <- sf.counties$NAME_2
```

```r
df.county.se <- matrix(, dim(sf.counties)[1], length(dates))
colnames(df.county.se) <- as.character(dates)
rownames(df.county.se) <- sf.counties$NAME_2

for (i in 1:dim(sf.counties)[1]) {
    pts.pred <- spsample(sf.counties[i, ], type = "regular", n = 1000)
    df.pred <- data.frame(pts.pred)
    names(df.pred) <- c("long", "lat")
    pts.pred.prism <- spTransform(pts.pred, CRS(projection(rs.precip)))
    df.pred$perc.grass.pasture.5000 <- extract(temp1, pts.pred)
    df.pred$perc.crop.5000 <- extract(temp2, pts.pred)
    temp3 <- df.pred

    for (t in 1:length(dates)) {
        temp4 <- data.frame(lat = temp3[, 2], long = temp3[, 1], perc.grass.pasture.5000 = temp3[,
            3], perc.crop.5000 = temp3[, 4], year = year(dates[t]), yday = yday(dates[t]))
        temp4$month <- month(dates[t]) + ifelse(year(dates[t]) == 2014, 0, ifelse(year(dates[t]) ==
            2015, 12, 24))
        x.precip <- get.temporal.x(rs.precip, pts.pred.prism, temp4$month, 1)
        temp4$precip.1 <- x.precip[, 1]
        temp4$precip.2 <- x.precip[, 2]
        x.tmean <- get.temporal.x(rs.tmean, pts.pred.prism, temp4$month, 1)
        temp4$tmean.1 <- x.tmean[, 1]
        temp4$tmean.2 <- x.tmean[, 2]
        temp4[is.na(temp4$tmean.1), 8:11] <- -1
        Xp <- predict(m.5000, type = "lpmatrix", newdata = temp4)
        a <- rep(1/dim(Xp)[1], dim(Xp)[1])
        Xs <- a %*% Xp
        df.county.prob[i, t] <- 1/(1 + exp(-Xs %*% coef(m.5000)))
        df.county.se[i, t] <- deltamethod(~1/(1 + exp(-x1)), Xs %*% coef(m.5000), (Xs %*% m.5000$Vp %*%
            t(Xs)), ses = FALSE)
    }
}

# Save small area estimates
write.csv(df.county.prob, file = "county estimates.csv")
write.csv(df.county.se, file = "county standard errors.csv")

# Standard errors of county-level estimates round(df.county.se,2)

# County-level estimates
round(df.county.prob, 2)
```

	2014-05-15	2014-06-15	2014-07-15	2015-05-15	2015-06-15
Barton	0.49	0.37	0.38	0.23	0.18
Dickinson	0.49	0.49	0.60	0.30	0.20
Ellis	0.62	0.53	0.52	0.39	0.36
Ellsworth	0.57	0.44	0.51	0.30	0.25
Finney	0.35	0.34	0.33	0.15	0.10
Geary	0.59	0.64	0.70	0.42	0.30
Greeley	0.25	0.27	0.34	0.10	0.07
Saline	0.54	0.48	0.60	0.30	0.21
Barton	0.76	0.75	0.80	0.45	0.36
Cape Girardeau	0.66	0.84	0.93	0.72	0.74
Cooper	0.70	0.87	0.90	0.71	0.65
Pettis	0.61	0.80	0.85	0.57	0.49
Pike	0.75	0.85	0.93	0.76	0.54
Stoddard	0.20	0.41	0.62	0.21	0.34
Cheyenne	0.33	0.31	0.49	0.10	0.08
Furnas	0.44	0.39	0.44	0.29	0.25
Hayes	0.46	0.39	0.55	0.27	0.23
Saunders	0.14	0.11	0.25	0.08	0.07
Bottineau	0.27	0.43	0.53	0.32	0.31
Ward	0.25	0.37	0.46	0.29	0.27
Hughes	0.72	0.80	0.89	0.75	0.61

```
Lake                 0.48       0.50       0.65       0.47       0.38
Tripp                0.66       0.74       0.84       0.66       0.54
Dallam               0.11       0.13       0.20       0.05       0.04
Randall              0.10       0.09       0.17       0.03       0.02
               2015-07-15 2016-05-15 2016-06-15 2016-07-15
Barton               0.60       0.41       0.51       0.77
Dickinson            0.60       0.52       0.58       0.86
Ellis                0.72       0.44       0.58       0.84
Ellsworth            0.68       0.44       0.53       0.83
Finney               0.41       0.31       0.40       0.53
Geary                0.71       0.59       0.69       0.92
Greeley              0.33       0.21       0.31       0.52
Saline               0.65       0.51       0.59       0.88
Barton               0.70       0.71       0.63       0.88
Cape Girardeau       0.82       0.84       0.86       0.97
Cooper               0.72       0.87       0.89       0.93
Pettis               0.65       0.83       0.84       0.92
Pike                 0.54       0.91       0.93       0.97
Stoddard             0.44       0.42       0.46       0.81
Cheyenne             0.45       0.37       0.40       0.76
Furnas               0.48       0.40       0.53       0.81
Hayes                0.57       0.41       0.54       0.85
Saunders             0.24       0.15       0.18       0.54
Bottineau            0.51       0.45       0.64       0.71
Ward                 0.44       0.38       0.62       0.70
Hughes               0.83       0.80       0.90       0.96
Lake                 0.52       0.55       0.62       0.87
Tripp                0.76       0.65       0.79       0.94
Dallam               0.13       0.13       0.15       0.29
Randall              0.12       0.15       0.17       0.31
```

References

Cressie, N. and Wikle, C. K. (2011). *Statistics for Spatio-temporal Data*. John Wiley & Sons.

Dormann, C. F., Elith, J., Bacher, S., Buchmann, C., Carl, G., Carré, G., Marquéz, J. R. G., Gruber, B., Lafourcade, B., Leitão, P. J., et al. (2013). Collinearity: a review of methods to deal with it and a simulation study evaluating their performance. *Ecography*, 36(1):27–46.

Gotway, C. A. and Young, L. J. (2002). Combining incompatible spatial data. *Journal of the American Statistical Association*, 97(458):632–648.

Khalaf, L., Timm, A., Chuang, W.-p., Enders, L., Hefley, T., and Smith, M. (2020). Data for: Spatio-temporal modeling of *Aceria tosichella* biotype occurrence and distribution.

Wood, S. N. (2017). *Generalized Additive Models: An Introduction with R*. CRC press, 2 edition.

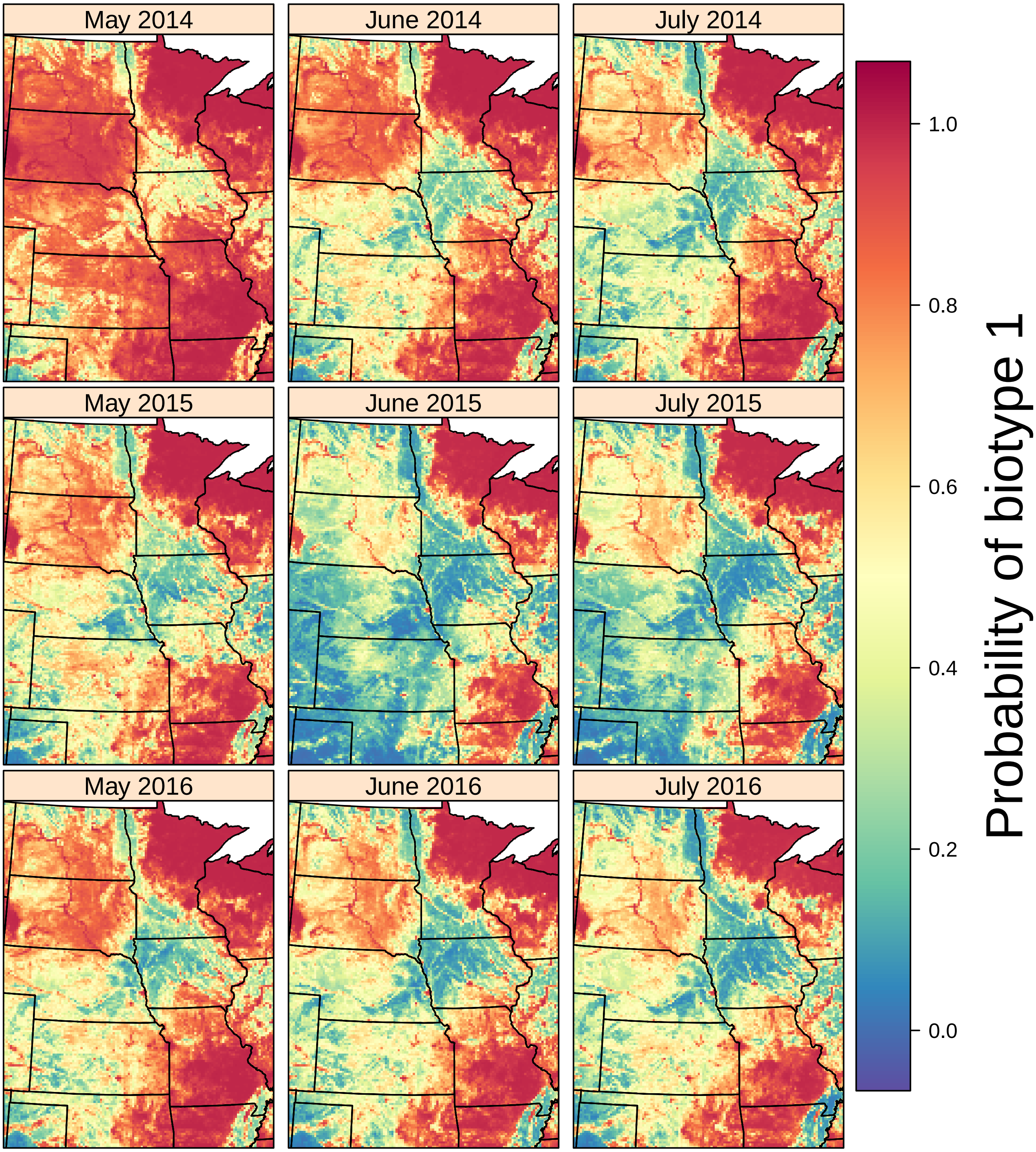

Figure 4. This predicted heat map was used to create figure 4 in the manuscript.
