# Supplementary material for: Modeling Aceria tosichella biotype distribution over geographic space and time
Source: PLoS One. 2020 May 29;15(5):e0233507. doi: 10.1371/journal.pone.0233507 (PMC7259573; doi:10.1371/journal.pone.0233507)
Supplement: S1 File — (RTF) [file pone.0233507.s012.rtf]

Contained within the folder To Mike (April 2 2020) is the following1). Reproducible analysis.pdf contains all the R code needed to reproduce the analysis. This should be submitted as supporting material with the publication. 2). Data.zip is a very large compressed folder that contains all of the data needed to reproduce the spate-temporal statistical analysis. This compressed folder should be submitted as supporting material with the manuscript. 
